# Cost of Maintenance of the Insane

**Published:** 1877-09

**Authors:** H. B. Wilbur


### MISCELLANEOUS.

### Cost of Maintenance of the Insane.

H. B. WILBUR, M. D.

In thirteen lunatic hospitals, containing some 2,000 pay-cases of the well-to-do classes, the average weekly cost was $7.42 (reduced to U. S. currency). These institutions correspond with a class of asylums in the United States like Bloomingdale, the Retreat at Hartford, Conn., the McLean Asylum at Charlestown, S. C.

Some of them have large endowments, as in the case of Bethlem Hospital, in which case more or less of the patients are supported gratuitously.

In the English county asylums which, in their mode of support and in their general scope, correspond with our State asylums, the average weekly cost of maintenance, including clothing, is less than three dollars; in similar asylums in Scotland and Ireland considerably less.

In comparing the cost of maintenance in the British institutions, with those of the same grade in the United States, several facts are to be borne in mind.

The salaries of officers are about the same in either case. The wages of attendants and servants are nearly seventy per cent. higher in the United States than in England. The staple articles of provisions cost much less in America. Looking at the bills of British asylums, I see that beef costs 24 cents per pound, mutton 20 cents, pork 17 cents. Of course, flour and grain are higher. Fuel is about the same.

One source of expense in American asylums, is the supposed necessity for the free use of stimulants and the extra diet required. In an institution, containing 600 patients, where pains had been taken to record the number of extra meals it is stated that over 17,000 were furnished in a single year. The question of employment of the insane has some relation even to this source of expense, if we may accept the opinion of Dr. Rutherford, of the Argyll and Bute asylum.

He thus remarks: "Insanity is, essentially, a disease of diminished vitality, and, when present, the system demands a stimulus; experience proves that there is no stimulant equal to active out-door employment and abundance of fresh air. The more this system is carried out, the plainer need be the food, and the fewer the extras required to maintain the standard of health, because the patients are brought more into the condition, and demand rather the fare of ordinary persons, than of lunatics kept under the irritating and depressing influences of forced confinement.

Taking fifteen of the English and Scotch asylums whose reports are before me, the ratio of attendants to patients is one attendant to eleven patients. The policy which governs generally, in the construction of asylums and in the scale of care and maintenance, is well expressed in the following extract from the last report of Dr. Lalor, Medical Superintendent of the Richmond County Lunatic Asylum, Dublin:

"As a large sanitary question, the best mode of providing for the insane *in thir own interest*, can, in my mind, receive a satisfactory practical solution only by dealing with it frym the point of view, of obtaining the best average result on the whole, all things being fairly considered. From this point of view, the advocacy of any expensive system for the care of the insane is, I think, not judicious. For the present, at least, the desires of the most humane philanthropic and benevolent, as regards insane, are most likely to be realized by some system which would bring all the insane of the empire under enlightened and kindly supervision, on some system capable of realization at a moderate cost. Much has been done in latter years in the three portions of the empire, in the way of providing increased asylum accommodation, and if the character of that accommodation has not been all that the advocates for individual treatment and for the highest order of internal comfort would desire; yet it should not be fogotten, that a greater number of persons have gained the advantage of a great improvement in their condition than could or would have been the case by more expensive arrangements, which, at the best, would have given higher results only to a smaller number of cases."

The proportion of insane in county poor-houses is somewhat less in Great Britain than in the United States. If the great preponderance of pauperism in the former country were taken into account, it might be said the proportion of the insane in county poor-houses is very much less.

Take, for example, Scotland, which, in point of population, is not unlike the State of New York, leaving out the cities of New York and Brooklyn. The percentage of insane in parochial asylums and in the lunatic wards of poor-houses in Scotland is 15 per cent. In the State of New York, 20 per cent.

In a late census of Scotland it is stated that very nearly one-third of the population live in houses of one room; much more than two-thirds live in houses of one or two rooms, while 82 per cent live in houses of three rooms and under.

These facts have been cited as, in some degree, a measure of the inability of the people of Scotland to naintain insane relatives in asylums, and will serve to show their great inferiority, in that respect, to the general population of the United States. Nevertheless, it should be borne in mind that of the number of insane to be found in Scotch poor-houses and parochial asylums, very few are of a character to require positive medical or other treament. Such cases are almost all cared for in some form of asylum specially designed for them and under competent medical supervision. And the reason why the civil authorities are enabled to make this general and adequate provision for the care of the insane at the public expense, may be gathered from the paragraph above quoted from the report of Dr. Lalor.

The provision made to meet their wants is so judiciously planned, and so economically administered, that the burden it involves is not too heavy for the shoulders of the tax payers. Palaces are not provided for a favored few of the indigent and pauper insane, and a large remainder consigned to neglect and suffering in inadequate quarters, but a comprehensive and well-adjusted system of relief is extended to all. The term palaces may, perhaps, be thought an extravagant one to apply to such institutions, till the facts are examined. But a three-storied structure, that is a quarter of a mile long and with a Mansard roof, certainly has a palatial appearance. Then look at the cost of some of these asylums. Two insane hospitals in Massachusetts are now in course of construction, the cost of which, as estimated by the Massachusetts Board of State Charities, is at the rate of $2.500 or $3,000 for each patient. One lately completed near Baltimore is on the same scale of expenditure.

The State of New York has now partially completed three large palaces for the insane that are a discredit to the social economy of any people. When completed they will have cost according to the estimates of governors and State comptrollers, about five millions of dollars, or at the rate of about $4,000 a patient, The plan of the Poughkeepsie asylum was conceived at Utica. It has been carried out thus far, by one of Dr. Gray's assistants. The asylum at Middletown was built to rival the other two. While the last one, now going up at Buffalo was exclusively planned by Dr. Gray, who has also been one of its building commissioners, from the outset. It was estimated to cost $800,000, to accommodate 500 patients. The city of Buffalo gave the land and brought the water supply to its very doors. Up to this time, the State has already appropriated $970,000, and the Commissioners in their last report ask for $200,000 more to render half the structure available. That this will not suffice is seen in the fact that in a proposed expenditure of $225,000 only $301 are allowed for contingencies. And if it should, what has this building committee to show for this vast outlay of public money? A lofty and pretentious center building and two wings, built of stone. These furnish quite palatial quarters for the officers and a third of the patients. The remainder will be housed in remoter wings of brick. Had the entire building been of the same material it would have been as well and at a great saving of cost. As it is, it is a mongrel structure with a long front, neither one thing nor another—an architectural abortion. The pretentiousness of a part, by contrast, degrades the appearance of the remainder. The walls are needlessly thick, preventing that insensible ventilation that is now recognized as an important mode of air-supply and at the same time giving a prison-like aspect to the patient's surroundings. The ceilings are so high as to render the proportions of the single rooms quite absurd and their ventilation difficult. This also involves stairways so high and intricate, that it will be no easy matter to get the patients out for labor or exercise in the open air. There are single dormitories for nearly all the patients, much increasing the cost; when a large percentage of them should be provided for in associated dormitories, as British experience fully shows. The workshops are utterly inadequate for the employment of the patients.

Five thousand dollars are to be expended for tram-ways to carry food to the several wards, and $18,000 for iron window-guards, when all the new Scotch insane asylums are absolutely without any provision for such a purpose. In fact, the whole establishment is so costly, so pretentious and at the same time so ill-adapted to the purpose for which it was designed, namely, the care and custody of the average insane, three-fourths of whom are to be indigent or pauper cases, that it will doubtless be known to the alienists of the next generation, as "Gray's folly."

All this extravagance, when it is known that the best modern asylums in England cost less than $1,000 a patient; and, as a rule, this includes land and furniture, no small items; while the difference in cost of labor and materials is now less than thirty per cent.

To my inquiries of one or more of these commissioners of the present cost of asylums for the insane, they gave as a maximum £150 per patient, including land and furniture. This, reduced to American currency, would be about $850 for each patient.

The Surrey County Asylum at Brookwood, known all over England as a model asylum, has just erected a new building for 300 patients that meets the entire approval of the commissioners. It is complete in itself, with kitchen and laundry, and all other domestic offices. After spending some hours in inspecting it, I could see no way in which it could be improved. This building, properly equipped and furnished, was completed at a cost of less than $750 per patient.

In New Jersey, a new asylum to accommodate 800 or 900 patients is nearly completed at an estimated cost of $3,000,000; or more than $3,000 a patient. The per capita cost in this last case would have been still more, had it not have been for an after-thought, that led to fitting up the attic story as wards for patients. Which after-thought, it may be mentioned in passing, involves the necessity of a tiresome journey up and down four flights of stairs at every time the patients in these upper wards take exercise in the open air.

Let it be remembered, in this connection, that the large majority of the patients, who are to occupy these buildings, are, physically, ailing rather than sick; that infection is not to be guarded against as in an ordinary hospital; that they need only proper custodial accommodations, which means a little more provision in the way of cubic space, heating and ventilating apparatus and special appliances, than is necessary for the health of the non-insane, and that their surroundings should not be too much at variance with those to which they have been accustomed.

It is not intimated in any quarter that the lage cost of any of the asylums, that have been referred to, is the result of any dishonest misappropriation of funds supplied for their erection, but a want of judgment in the plans adopted, local pride, professional ambition and facility of securing State appropriations will explain it all.

Extravagant outlay in constuction too often involves a corresponding increase in the current expenses of the establishment.

At the three State Lunatic Hospitals of New York, now containing 911 patients, the average weekly cost of maintenance is more than six dollars.

If such weekly cost were reduced to four dollars—and the weekly cost of maintenance at the three Massachusetts institutions of a similar grade is less than four dollars; and, at the Willard Asylum for Chronic Insane, in this State, but a little more than three dollars--450 more insane persons could receive adequate care and treatment without additional expense to the tax payer.

Had we a Board of Lunacy, as in England, would not the public mind be awakened to this fact? And would not the effort be made to reduce the scale of expenditure in our hospitals to that of corresponding institutions in other States?—*H. B. Wilbur, M. D., in his Report on the Management of the Insane in Great Britain.*

